# Interactions Among Circadian Rhythms, Aging, and Neurodegenerative Diseases

**DOI:** 10.1002/alz70855_096381

**Published:** 2025-12-23

**Authors:** Shampa Ghosh, Krishna Kumar Singh, Prashant Verma, Amit Kumar Pandey, Jitendra Kumar Sinha

**Affiliations:** ^1^ GloNeuro Academy, Noida, Uttar Pradesh, India; ^2^ Symbiosis International (Deemed University), Pune, Maharastra, India; ^3^ BML Munjal University, Gurugram, Haryana, India; ^4^ Amity University Uttar Pradesh, Noida, UP, India

## Abstract

**Background:**

Circadian rhythms are critical homeostatic mechanisms in biological systems, regulating a wide range of physiological processes including metabolism, endocrinology, and cognitive functions. In most individuals, these rhythms are significantly altered with aging, such as reduced variability in sleep‐wake cycles and changes in their timing. These disruptions can accelerate age‐related physiological decline, which is associated with increased oxidative stress and inflammatory responses. It is assumed that relationships between circadian mechanisms and aging processes determine the onset and progression of neurodegenerative disorders.

**Method:**

This work synthesizes current research on the physiological role of circadian rhythms and their modulation with advancing age. It examines how age‐associated circadian disruptions contribute to neurodegenerative diseases by altering metabolic and physiological pathways. The focus is on reviewing literature that connects circadian health with neurodegeneration through biological clocks' regulation of redox balance and other metabolic functions.

**Result:**

The evidence from research shows that age‐related disruptions in circadian rhythms not only predispose individuals to neurodegenerative diseases but also promote the rate of disease progression. This disruption leads to deregulation in antioxidative and inflammatory responses, affecting neuronal health and functionality. Age‐related changes in circadian control are known to affect cognitive and emotional behaviors, critical in neurodegenerative diseases like Alzheimer's and Parkinson's disease.

**Conclusion:**

Understanding complex relationships of circadian rhythmicity with the process of aging is essential in designing adequate interventions to delay the emergence and severity of neurodegenerative diseases. Through this review, it should be possible to identify pathways where circadian disruption intervention could be valuable in allowing healthier aging to prevent, at least partially, or even delay onset or development of neurodegenerative diseases. Further studies into circadian biology in the context of aging could open the gates for novel preventions and therapy that enhances life quality in elderly populations.

